# Less major bleeding and higher hemoglobin after left atrial appendage closure in high‐risk patients: Data from a long‐term, longitudinal, two‐center observational study

**DOI:** 10.1002/clc.24123

**Published:** 2023-08-13

**Authors:** Christian Schach, Raphael Reitschuster, Dennis Benedikt, Elias Füssl, Kurt Debl, Lars S. Maier, Andreas Luchner

**Affiliations:** ^1^ Department for Internal Medicine II University Heart Center Regensburg Regensburg Germany; ^2^ Department for Cardiology Hospital Barmherzige Brüder Regensburg Regensburg Germany

**Keywords:** anticoagulation, atrial fibrillation, bleeding, hemoglobin, left atrial appendage closure, long‐term longitudinal observation

## Abstract

**Background:**

Left atrial appendage closure (LAAC) is a mechanical alternative for stroke prevention in patients at risk who cannot tolerate oral anticoagulation (OAC).

**Hypothesis:**

Our hypothesis was that the reduction of anticoagulation following LAAC results in a decrease of bleeding events and a rise in serum hemoglobin in a high‐risk collective of patients with atrial fibrillation (AF).

**Methods:**

Bleeding events, use of erythrocyte concentrates, anticoagulation, embolic events, and serum hemoglobin levels before and following LAAC were compared over more than 4 years.

**Results:**

Seventy‐five patients (CHA₂DS₂‐VASc score 4.4 ± 1.7, HAS‐BLED score 4.6 ± 1.1) were analyzed. Before LAAC (observation period 1.8 ± 1.8 years), 67 patients experienced 1.8 ± 1.4 bleeding events (0.9 ± 1.3 major) per year resulting in 0.7 ± 1.3 transfusions per year. After LAAC (2.6 ± 2.0 years), 26 patients (*p* < .0001 vs. before) had 0.6 ± 2.1 bleeding events (*p* < .0001), 0.2 ± 0.6 major bleedings (*p* < .0001) and received 0.6 ± 1.9 transfusions per year (*p* = .671). Fourteen patients had stroke before and 3 after LAAC (*p* = .008). Serum hemoglobin increased from initially 9.9 ± 3.0 to 11.9 ± 2.3 g/dL until the end of follow‐up (*p* = .0005). Adverse embolic events did not differ before and after LAAC in our collective.

**Conclusion:**

In this clinical relevant cohort of AF patients with high risk for stroke and intolerance to OAC, we show that LAAC was able to reduce the rate of stroke and bleeding events, which translated into a rising serum hemoglobin concentration.

## Graphical abstract

In patients with atrial fibrillation (AF) at high risk for stroke and bleeding events, closure of the left atrial appendage (LAAC) led to a relevant reduction of bleeding events and stroke as well as an increase in serum hemoglobin. RRR, relative risk reduction; TIA, transitory ischemic attack.

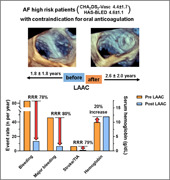

## 1 INTRODUCTION

Atrial fibrillation (AF), together with the presence of cardiovascular risk, clearly amplifies the probability of ischemic stroke, also represented by the CHA₂DS₂‐VASc score.^1^ For patients at risk of stroke, oral anticoagulation (OAC) is the standard prevention therapy. In the presence of contraindications to OAC, however, left atrial appendage closure (LAAC) is a mechanical substitute for stroke prevention in patients with AF.^2^, ^3^, ^4^, ^5^ Current guidelines state that LAAC may reduce stroke risk in patients with contraindications to OAC, yet note that the implantation procedure can also cause serious complications with real‐world event rates being possibly higher than those of industry‐sponsored trials.^6^ The most common complications are bleeding, pericardial effusion, access‐site related, or device related thrombus, possibly leading to stroke.^7^, ^8^ Regarding the latter, it is important to mention that in large trials, that is, PROTECT AF and PREVAIL, ischemic stroke tended to be higher after LAAC compared to OAC and the non‐inferiority of LAA occlusion was mostly driven by the prevention of hemorrhagic stroke.^5^, ^6^ Thus, the ideal indication for LAAC is bleeding diathesis in combination with risk of stroke in the presence of AF (i.e., elevated CHA₂DS₂‐VASc score).

In our current study, we sought to evaluate common efficacy and safety endpoints under real‐world and all‐comer conditions. Our study comprises a particular long periinterventional period and compares clinical events and laboratory changes from long before until long after the LAAC procedure. It demonstrates reliable stroke prevention of LAAC and important additional benefits through reduction of bleeding events and increase in serum hemoglobin after the procedure.

## 2 MATERIALS AND METHODS

### 2.1 Study design

This observational registry of patients with nonvalvular AF and contraindication to OAC assessed the safety and efficacy of LAAC at two cardiologic centers in Germany (University Hospital Regensburg and Hospital Amberg). For LAAC, commercially available devices (Amplatzer, Abbott and Watchman, Boston Sci.) were employed. Inclusion criteria were age >18 years, paroxysmal, persistent, or permanent nonvalvular AF, contraindication to OAC and CHA₂DS₂‐VASc score of ≥1 (gender not considered). Exclusion criteria included evidence of an intracardiac thrombus, active infection, and LAA anatomy unable to accommodate a device per sizing guidelines. Patients were considered enrolled in the study following consent and introduction of the device delivery system to the vasculature. Due to the longitudinal approach of this observational prospective registry, no matching was necessary.

### 2.2 LAAC

After evaluation and sizing of the left atrial appendage (LAA) via transesophageal echocardiography (TEE) and contrast media injection into the LAA, devices in the diameter range from 18 to 31 mm—varying on LAA morphology—were chosen. The implantation was guided by fluoroscopy and TEE to verify proper positioning and stability. After implantation, patients were treated with dual antiplatelet therapy (DAPT) for 3 months until the first control visit.

### 2.3 Echocardiographic assessment

TEE was undertaken before the procedure to rule out thrombus and assess LAA size and shape. TEE guided the procedure and a transthoracic echocardiogram was acquired predischarge to assess device location and check for pericardial effusion. TEE was again performed 3 months after implantation. The TEE protocol included omniplane views of 0°, 60°, and 110° to obtain information regarding LA/LAA thrombus, LAA ostium size, length distal to the LAA ostium, peri‐device flow, and procedural complications such as device embolization and pericardial effusion. Evaluation of the occluder position was conducted by assessing complete occlusion, residual peri‐device flow, and device compression.

### 2.4 Clinical events

Clinical events for this analysis were bleeding, red blood cell (RBC) transfusion, and stroke. A major bleeding was defined as an event meeting Bleeding Academic Research Consortium (BARC) ≥3. Type 3a is a drop in hemoglobin (≥3 to <5 g/dL) with overt bleeding requiring transfusion, 3b is overt bleeding with a hemoglobin drop ≥5 g/dL, cardiac tamponade, or bleeding requiring surgical intervention, 3c is intracranial hemorrhage or intraocular bleed compromising vision, and type 5 is probable or definite fatal bleeding. Clinical events before LAAC were evaluated at admission and periprocedural events at the time of discharge. Following events were acknowledged during follow‐up.

### 2.5 Follow‐up visits

Assessment of medication and events was performed at 3 and 6 months after LAAC, additionally if events occurred, and furthermore by telephone call if implantation occurred at least 1 year ago.

### 2.6 Hemoglobin concentration

Hemoglobin concentration was assessed before and after LAAC: Presence of AF in patients with anticoagulation and clinical adverse bleeding events commonly triggers consultation potentially leading to LAAC. Hemoglobin values before as well as during follow‐up were acquired from referring doctors or hospitals involved in the patients treatment.

### 2.7 Ethical approval

Use of retrospectively anonymized patient data is covered by the Bavarian hospital act. Requesting further information by phone call to the patient or to the referring doctor is covered by the local ethics committee (Ethics committee at University of Regensburg, approval number 18‐928‐101). We adhere to the statement of ethical publishing as appears in.^9^

### 2.8 Statistical analysis

Continuous variables are presented as mean ± SD, categorical variables are presented as frequencies and percentages, while further descriptive analysis was conducted using *χ* ^2^ analysis. For comparison of longitudinal data before and after LAAC, paired *t*‐testing was applied. All statistical analyses were performed with Prism version 9 (Graphpad). Statistical significance was assumed when the null hypothesis could be rejected at *p* < .05.

## 3 RESULTS

### 3.1 Study population

Seventy‐five patients (mean age 75 ± 8 years, 36% female) were analyzed. The study flow chart is presented in Supporting Information: Figure 1, baseline characteristics are listed in the Supporting Information: Table 1. During follow‐up, 66 patients (88%) received TEE for device evaluation.

### 3.2 Risk for bleeding and stroke/TIA

**Figure 1:**
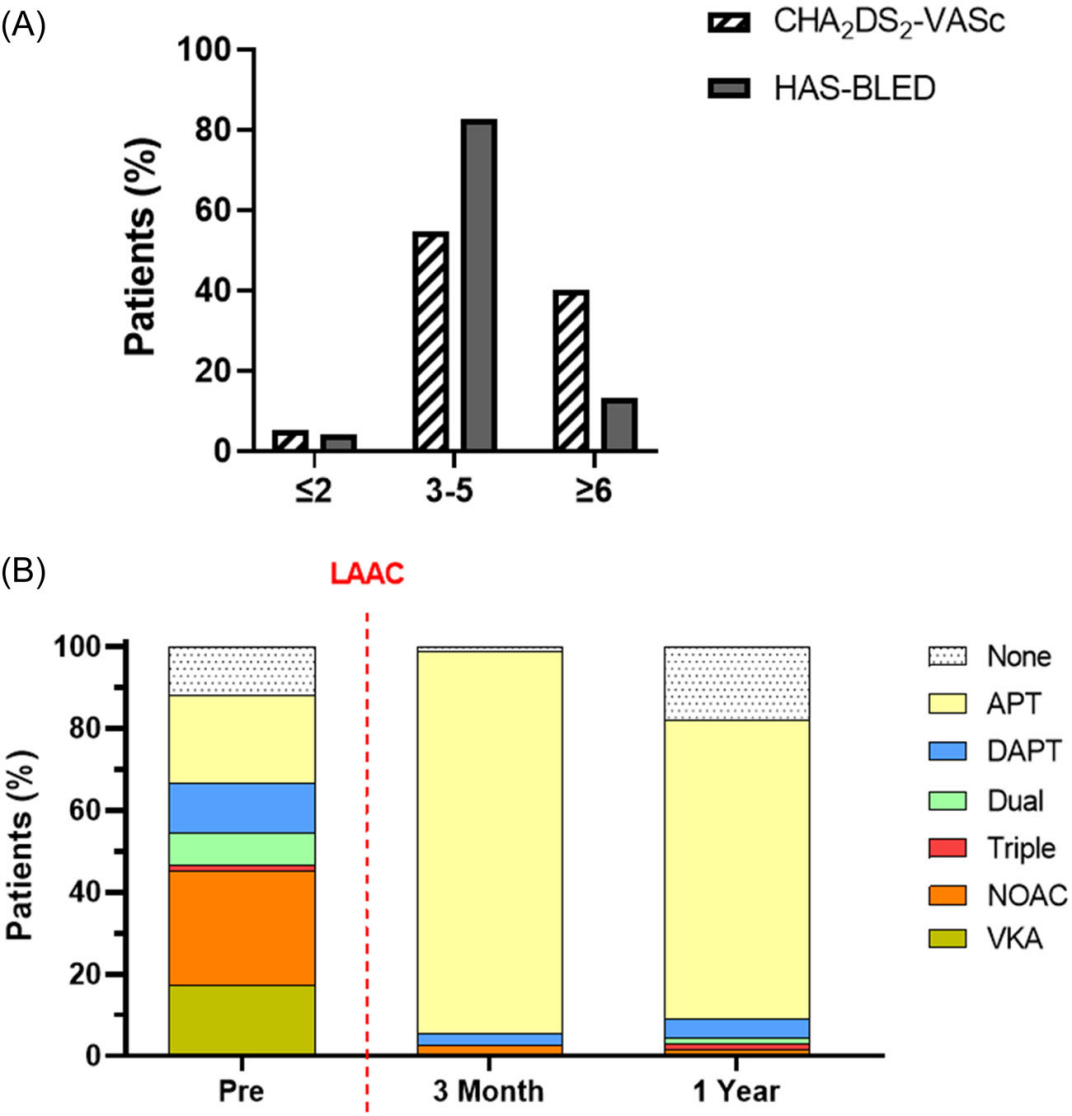
Risk for stroke and bleeding our real‐world collective. (A) Frequency of patients with scores for stroke (CHA_2_DS_2_‐VASc) and bleeding (HAS‐BLED) risk. For the calculation of the CHA_2_DS_2_‐VASc score, gender was not considered. (B) Anticoagulation strategy applied before, 3 months and 1 year after LAAC. APT, antiplatelet therapy; DAPT, dual antiplatelet therapy; LAAC, left atrial appendage closure; NOAC, non vitamin K dependent oral anticoagulation; VKA, vitamin K antagonist.

Patients, on average, were at high risk of stroke with a CHA_2_DS_2_‐VASc score of 4.4 ± 1.7 and bleeding with a HAS‐BLED score of 4.6 ± 1.1, corresponding to an annual stroke risk of 7% and an annual major bleeding risk of 8%.^10^, ^11^ Ninety‐five percent of the patients analyzed in this study had a CHA_2_DS_2_‐VASc score ≥3 and 40% a score ≥6 (Figure 1A), representing a collective with high risk of stroke or TIA. On the other hand, 96% of the patients had a HAS‐BLED score of ≥3 and 13% of ≥6. The mean HAS‐BLED score of 4.6 underlines the high risk for bleeding in our cohort, as in a COX regression analysis a score >2 revealed an 85% increase of risk for any clinically relevant bleeding in patients with AF and anticoagulation.^12^ These risk scores are influenced by the medication. Before LAAC, 54% of patients received non vitamin K dependent oral anticoagulants (NOAC) or vitamin K antagonists (VKA), and 9.3% of them also received APT. This compares with 94% of patients who received APT alone or nothing 3 months after LAAC and 91% at 1 year (Figure 1B).

### 3.3 Clinical endpoints before and after LAAC

**Figure 2:**
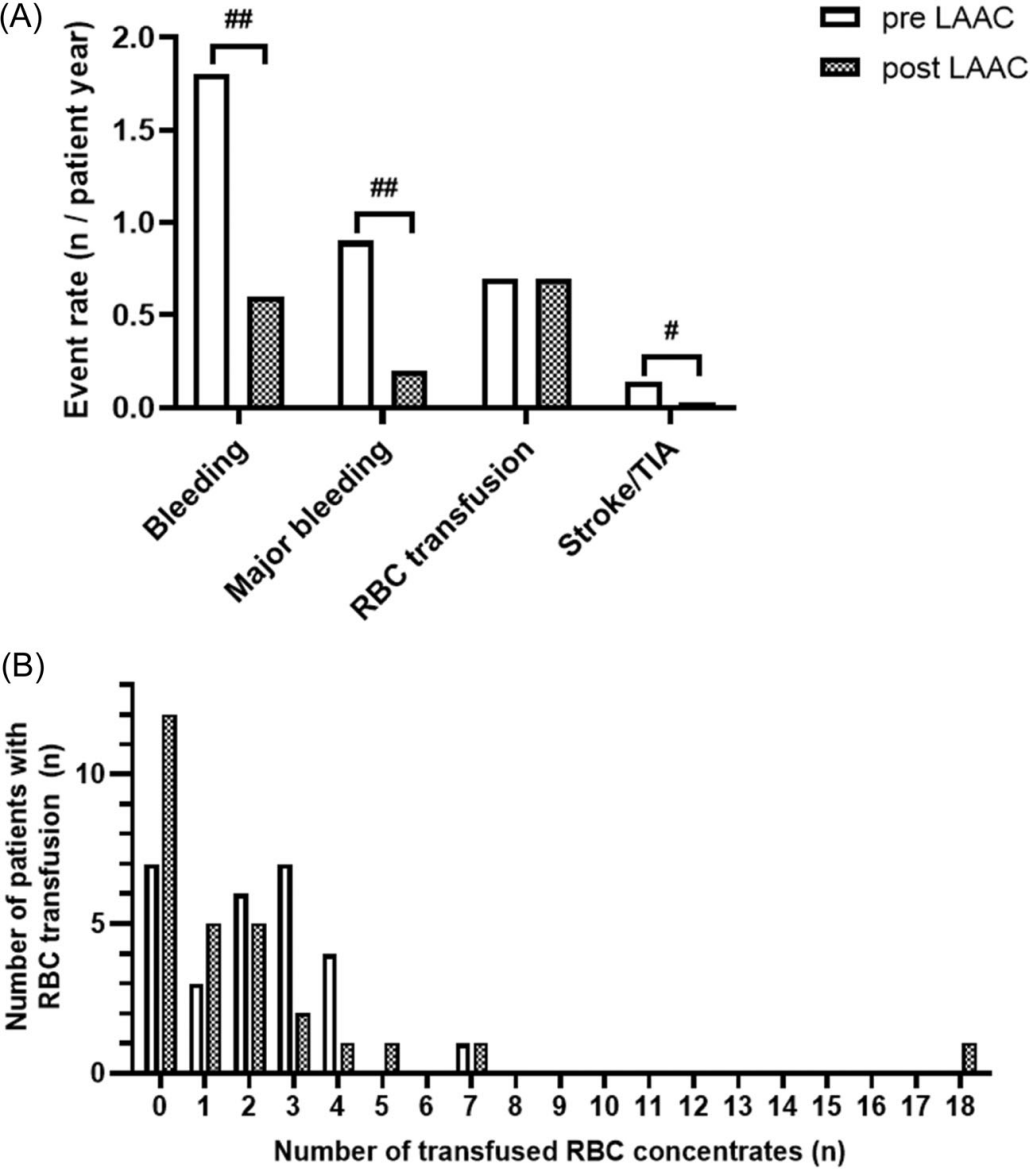
Event rates of clinical endpoints and use of RBC concentrates. (A) Event rates before (pre‐LAAC) and after (post‐LAAC) were compared in a longitudinal manner. (B) Frequency distribution of the number of transfused RBC units per patient before (pre‐LAAC) and following (post‐LAAC). ^#^ *p* < .01, ^##^ *p* < .0001, paired student's *t*‐test. LAAC, left atrial appendage closure; RBC, red blood cell.

Although the observational period after LAAC was longer, the number of stroke or TIA was less after LAAC (Figure 2A and Table 1): 14 patients suffered from stroke before LAAC and 3 patients following LAAC. At the time of stroke, 4 patients each received APT, anticoagulation with VKA, or no blood thinning therapy; 1 patient received DAPT and 1 patient received NOAC (Supporting Information: Figure 2). With respect to bleeding, we observed less events including major bleeding: Before LAAC, 67 patients experienced 1.8 ± 1.4 bleeding events (0.9 ± 1.3 major) per year. After LAAC, 26 patients had 0.6 ± 2.1 bleeding events (*p* < .0001) and 0.2 ± 0.6 major bleedings (*p* < .0001). However, neither the number of RBC transfusions nor the number of patients who received RBC transfusions considerably differed. This was mostly due to 3 patients, who experienced severe gastrointestinal bleeding after LAAC (compare Supporting Information: Table 2, in which all patients with bleeding events post‐LAAC were characterized). The rates of bleeding and transfusion as well as the distribution of transfused RBC are depicted in Figure 2. Adverse periprocedural or postprocedural events are listed in the lower half of Table 1.

**Table 1:** Clinical endpoints before versus following LAAC.

	Prior	After	*p*	ARR	RRR
Observation period (years)	1.8 ± 1.8	2.6 ± 2.0	.007		
Cumulative events					
Bleeding, all	159	35	<.0001	165.3	78.0
Gastrointestinal	113	21		122.7	81.4
Nasal/oral	0	8		−10.7	na
Other	46	6		53.3	87.0
Major bleeding	83	17	<.0001	88.0	79.5
RBC transfusion	59	55	.815	5.3	6.8
Patients with					
Bleeding events	67	26	<.0001	54.7	61.2
Major bleedings	36	12	<.0001	32.0	66.7
RBC transfusions	21	16	.254	6.7	23.8
Stroke/TIA	14	3	.007	14.7	78.6
Periprocedural events (<7 days post‐LAAC)					
Pericardial effusion	na	4		na	na
Pericardial tamponade	na	2		na	na
RBC transfusion	na	2		na	na
Hemoptysis	na	1		na	na
Inguinal hematoma	na	1		na	na
Postprocedural events					
Device‐related thrombus	na	0		na	na
Device embolism	na	0		na	na

*Note*: Cumulative events and the number of patients with events were compared before and after LAAC. Stroke/TIA occurred once per patient. *p* Value calculation was assessed by paired student's *t*‐testing or *χ* ^2^.

Abbreviations: ARR, absolute risk reduction; LAAC, left atrial appendage closure; RBC, red blood cell; RRR, relative risk reduction; TIA, transitory ischemic attack.

### 3.4 Effect of LAAC on RBC transfusion and serum hemoglobin concentration

**Figure 3:**
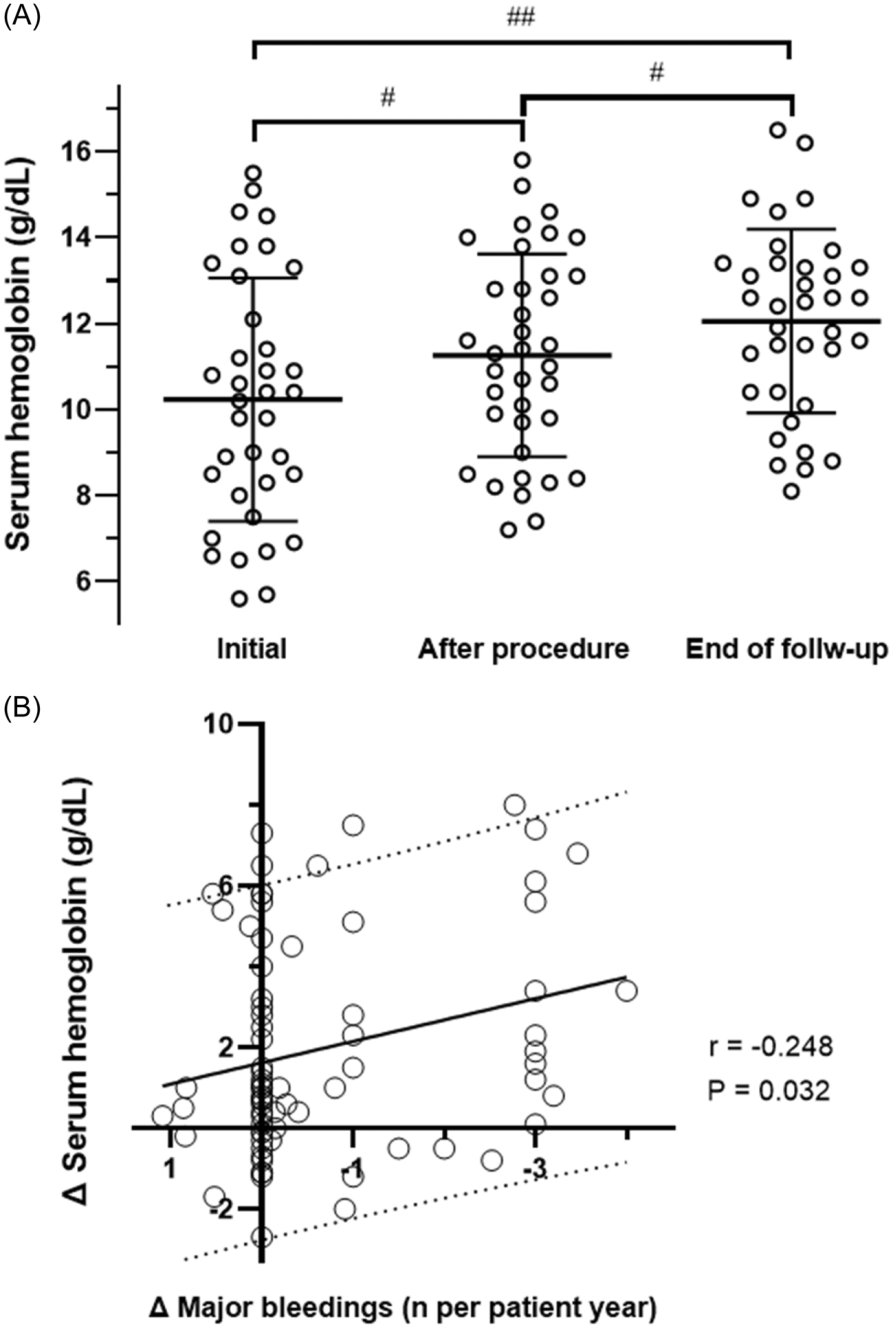
Longitudinal concentration of serum hemoglobin. (A) Scatter plots showing the temporal change of the serum hemoglobin concentration. Initial: first value in the observational period; after procedure: value at discharge after LAAC; end of follow‐up: last value during observational period. Two‐way ANOVA: *p* < .0001, paired student's *t*‐test: ^#^ *p* < .01, ^##^ *p* < .001. (B) Simple linear regression and correlation analysis of the difference in annualized major bleeding events (major bleeding events per patient year after LAAC minus major bleeding events per patient year before LAAC) versus the difference in serum hemoglobin values (serum hemoglobin at the end of the observation period minus serum hemoglobin at the beginning of the observation period). Solid line is best‐fit line, dashed lines are 90% prediction bands of the best‐fit line. LAAC, left atrial appendage closure; P, *p* value; r, Pearson's r.

Before LAAC, 21 patients received RBC transfusions (on average 0.8 ± 1.4); while following LAAC 16 patients received RBC transfusions averaging 0.7 ± 2.4. The median decreased from 2 to 1 transfused RBC unit, however, 1 patient received 18 units after LAAC (Figure 2B). The 3 patients with the biggest need for RBC transfusions after LAAC received 30 RBC units which equals 55% of the total transfused units. These 3 patients all had recurrent gastrointestinal bleedings (Supporting Information: Table 2). Serum hemoglobin concentration before LAAC ranged from minimal 4.6 g/dL to maximal 17.0 g/dL with a median of 9.8 (IQR 7.2−12.1) g/dL (for mean ± SD please see Figure 3A). Short term after LAAC, hemoglobin concentration had increased on average by 1.1 ± 2.2 g/dL compared to the first value within the observation period. From this point until the end of follow‐up it increased by another 0.9 ± 1.8 g/dL, resulting in a value of 11.9 ± 2.3 g/dL. Further, the deltas (before vs. after LAAC) of major bleedings events and serum hemoglobin levels correlated significantly (Figure 3B).

## 4 DISCUSSION

Patients with AF who are at high risk of stroke and bleeding represent a vulnerable and therefore important clinical cohort that is insufficiently captured by large‐scale randomized trials. In our long‐term observation of such patients, stroke rate, bleeding rate, and major bleeding rate decreased after LAAC, whereas serum hemoglobin increased. While large pivotal trials suggest that LAAC may be less effective than OAC in preventing ischemic stroke on trend, our data suggest that, in a bleeding‐prone population, in which anticoagulation is often inadequate, the mechanical option by LAAC may even be more effective than anticoagulation for stroke prevention.

### 4.1 A clinical relevant cohort: High risk of stroke plus high risk of bleeding

As the decrease in the risk of stroke is at the cost of an increased risk of bleeding, the use of anticoagulant agents is of limited clinical benefit if patients are prone to bleeding or are intolerant to these agents. The lifetime risk for AF is 37% in European ancestry at index age of 55 years^6^, ^13^ and more than 5% of AF hospitalizations were associated with gastrointestinal bleeding,^14^ indicating a relevant clinical problem. In the ORBIT‐AF II registry, the rate of major bleeding was similar in VKA and NOAC‐treated AF patients. However, NOAC‐related bleeding events required less blood product administration.^15^ Although the median rate of major bleeding in randomized clinical trials seems to be similar to observational studies for patients with AF receiving VKA,^16^ the risk for bleeding events is not consistently reported. In the ORIBT‐AF II study quantified bleeding risk by assessing the the Atria score, which has less predictive accuracy than the HAS‐BLED score,^17^ and the ORBIT bleeding score, which showed no better performance in predicting major bleeding events in anticoagulated AF patients.^18^, ^19^ Both scores show that >60% of patients in the ORIBT‐AF II study had a low bleeding risk, whilst in our study 96% had an HAS‐BLED score ≥3 equaling a high risk for bleeding. In PROTECT AF and PREVAIL, the bleeding risk score HAS‐BLED was not prospectively captured. The modified HAS‐BLED score, which was was calculated for the 5‐year follow‐up,^5^ indicated a high bleeding risk in 20% of the patients in PROTECT AF and 30% in PREVAIL. Also the HAS‐BLED score in PRAGUE‐17,^20^ which analyzed high‐risk patients with AF, was lower than in the the actual study, emphasizing the elevated bleeding risk in our cohort. The risk of stroke, represented by the CHA_2_‐DS_2_‐VASc score, is slightly lower than in PRAGUE‐17 (4.7) and higher than in the initial landmark studies PROTECT AF and PREVAIL (3.6 for the combined cohort) reflecting a comparable risk of stroke for our all‐comer cohort.

### 4.2 Reduction of stroke and bleeding events following LAAC

Previous randomized trials demonstrated a reduction of non‐procedure‐related major bleeding events and hemorrhagic stroke in patients treated with LAAC compared to warfarin.^5^ The rate of ischemic strokes did not differ significantly between these groups. VKA have widely been used as the primary choice of OAC in AF‐related stroke prevention and still have an edge in patients with kidney disease.^21^ NOACs, which cannot yet be used broadly in patients with kidney disease, also showed reduced major bleeding events.^22^ In comparison to NOAC, LAAC performed inconsistently: The procedure was associated with a higher risk for major bleeding in a study done by Noseworthy et al.^23^ and no difference in clinically significant bleeding was observed in the PRAGUE‐17 trial.^20^ Both studies did not show differences in ischemic stroke/systemic embolism for both treatment groups, thus it is questionable if LAAC provides net benefit for patients with AF with additional risk of stroke. In our study, more patients were on NOAC than on VKA before LAAC, reflecting a contemporary real world collective. Comparison of the bleeding events in our study before LAAC with the control arm of the combined PROTECT AF/PREVAIL cohort reveals an approximately 20‐fold higher rate (0.61 events per year) for the current study population. After LAAC, the major bleeding rate decreased to 0.09 per patient per year, which is still 3‐ to 4‐fold higher than in the combined cohort in.^5^ This is coherent with the high bleeding risk of those populations as mentioned above. Ischemic events were rare in our study, which is in line with various studies in literature.^3^, ^24^, ^25^

As mentioned in the introduction, use of RBC transfusions may not only provide beneficial effects. Surprisingly, we could not observe a decrease of transfusions after LAAC despite less bleeding/major bleeding events. Still, there were 12 patients who required transfusions before LAAC and managed without any after LAAC. During the observation period, most patients after LAAC received 1−2 RBC concentrates, whereas before LAAC most had 2−4. One patient (after LAAC) was an outlier with 18 RBC concentrates and thereby affected statistical analysis (compare Figure 2B). Due to necessary resources and potential adverse effects of RBC transfusions, we consider reporting the number of used RBC concentrates important in this context.

### 4.3 Serum hemoglobin increases after LAAC

Although we could not provide a statistically significant reduction of used RBC concentrates after LAAC, we witnessed an increase in serum hemoglobin levels, which could be explained by the diminished rate in major bleeding events. Although hemoglobin levels are implied in the definition of major bleeding events (e.g., BARC), hemoglobin levels have not consistently been reported before. Periprocedural hemoglobin concentration has been shown to decrease around LAAC,^26^ but to our knowledge no study has evaluated hemoglobin levels during a multiyear observational period so far. Thus, this study adds important data and shows a reduction of stroke and bleeding events after LAAC in high‐risk patients.

## 5 LIMITATIONS

The retrospective structure of the study implies a bias in data collection; indeed, five patients could not be analyzed due to missing data. Another limitation is the lack of a control cohort. However, since our total observation period covers intervals of almost equal length before and after the intervention (LAAC), each patient served as his own control. We assume that such an intra‐individual comparison renders the current results at least as reliable than those of a matched control cohort.

We found that the number of transfused RBC did not differ significantly before and after LAAC. This neutral finding might be biased by temporal trends in the indication of transfusions, which became more restrictive during recent years.^27^ We assume that the transfusion indication has been restrictive for a longer time than the observation period of our current study and that it is therefore unlikely that such a temporal trend should have had a relevant impact on our results. Further, our robust finding of elevated hemoglobin after LAAC persists and would become even more remarkable with a more restrictive transfusion indication after LAAC, indicating an even stronger anti‐bleeding effect of the current procedure.

## 6 CONCLUSIONS

In patients with AF who are at high risk of stroke and bleeding and who have a contraindication to OAK, LAAC has been shown to be a safe and reliable method to reduce bleeding and ischemic events in this longitudinal observational study. Consecutively, there was an increase in serum hemoglobin concentration; however, the number of transfused RBC units did not differ significantly after LAAC. One patient required an enormous number of RBC units during follow‐up on DAPT, suggesting a cautious indication for therapies requiring more than standard (mono)antiplatelet agents.

## AUTHOR CONTRIBUTIONS

*Conceptualization*: Christian Schach and Andreas Luchner. *Methodology*: Kurt Debl and Andreas Luchner. *Validation*: Kurt Debl, Andreas Luchner, and Christian Schach. *Formal analysis*: Dennis Benedikt, Raphael Reitschuster, and Christian Schach. *Investigation*: Dennis Benedikt, Raphael Reitschuster, Elias Füssl, and Christian Schach. *Resources*: Lars S. Maier and Andreas Luchner. *Data curation*: Christian Schach and Raphael Reitschuster. *Writing—original draft preparation*: Christian Schach and Raphael Reitschuster. *Writing—review and editing*: Raphael Reitschuster, Dennis Benedikt, Andreas Luchner, Kurt Debl, and Christian Schach. *Visualization*: Raphael Reitschuster and Christian Schach. *Supervision*: Andreas Luchner and Lars S. Maier. *Project administration*: Christian Schach. All authors have read and agreed to the published version of the manuscript.

## CONFLICT OF INTEREST STATEMENT

The authors declare no conflict of interest.

## Supporting information

Supporting information.

## Data Availability

The data presented in this study are available from the corresponding author upon reasonable request.
