# Supplementary material for: Less major bleeding and higher hemoglobin after left atrial appendage closure in high‐risk patients: Data from a long‐term, longitudinal, two‐center observational study
Source: Clin Cardiol. 2023 Aug 13;46(11):1337–44. doi: 10.1002/clc.24123 (PMC10642336; doi:10.1002/clc.24123)
Supplement: Supplementary file 1 — Supporting information. [file CLC-46-1337-s001.docx]

**Supplemental Material**


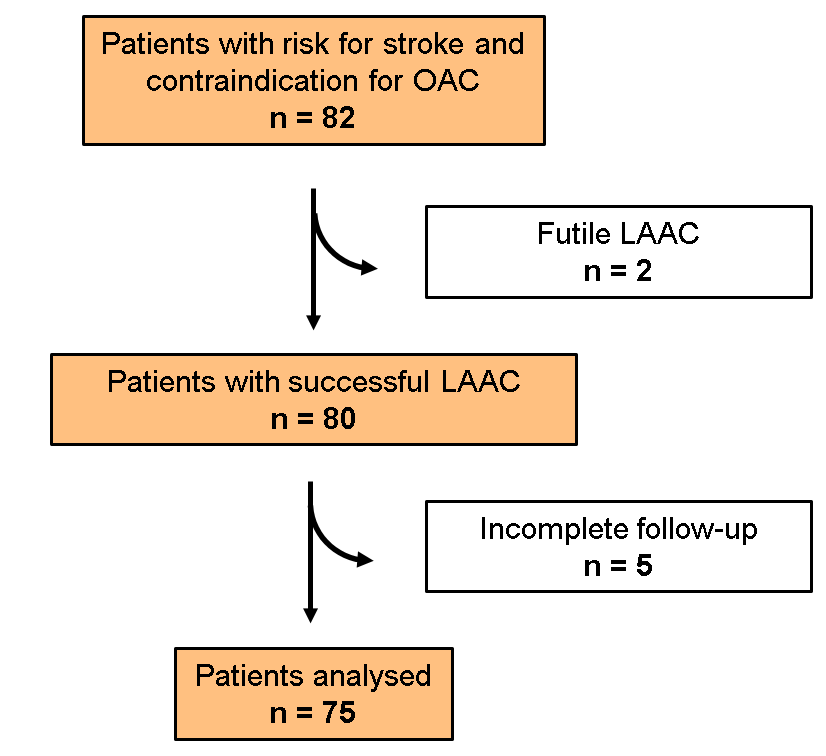


**Supplemental Figure 1.** Study flow chart. Patients with AF, CHA_2_DS_2_-VASc ≥1 and contraindication to oral anticoagulation (OAC) were included. In 2 patients, LAAC was not possible and in 5 patients, follow-up was incomplete, thus 75 patients were analyzed. After LAAC, 88% received transesophageal echocardiography (TEE) during follow-up.

**Supplemental Table 1.** Baseline characteristics.

| **Characteristic** | **Patients (n = 75)** |
| --- | --- |
| Age at procedure, y | 74.9 ± 7.6 |
| Age >75 | 40 (53.3) |
| Female gender | 27 (36) |
| LVEF <50% | 21 (28) |
| Vascular disease | 42 (56) |
| Abnormal renal or liver function | 42 (56) |
| Arterial hypertension | 67 (89.3) |
| Diabetes mellitus | 21 (28) |
| CHA_2_DS_2_-VASc score | 4.4 ± 1.7 |
| HAS-BLED score | 4.6 ± 1.1 |

Values are mean ± SD or n (%). Abbreviations: NYHA = New York Heart Association; LVEF = left ventricular ejection fraction.

**Supplemental Table 2**. Characteristics of patients with bleeding events after LAAC.

|  |  |  |  |  | Pre LAAC | | | | | Post LAAC | | | | |  |  |  |
| --- | --- | --- | --- | --- | --- | --- | --- | --- | --- | --- | --- | --- | --- | --- | --- | --- | --- |
| **No.** | **Age**  **(y)** | **Gender** | **CHA₂DS₂-VASc** | **HAS-BLED** | **Bleeding**  **events** | **Major**  **bleedings** | **RBCT** | **ACT** | **Bleeding**  **events** | | **Major**  **bleedings** | **Time**  **(days)** | **RBCT** | **ACT** | **Diagnosis** | | |
| 1 | 66 | m | 2 | 5 | 4 | 0 | 0 | 1 | 1 | | 0 | 4 | 0 | 1 | Recurrent GI bleedings | | |
| 2 | 80 | m | 6 | 4 | 2 | 2 | 7 | 3 | 6 | | 5 | 29 | 18 | 1 | Recurrent GI bleedings with malaena, colon diverticulosis, small bowel telangiectasia | | |
| 3 | 77 | f | 5 | 5 | 3 | 3 | 4 | 3 | 1 | | 1 | 25 | 2 | 1 | [Subconjunctival hemorrhage](https://en.wikipedia.org/wiki/Subconjunctival_hemorrhage) | | |
| 4 | 61 | m | 2 | 3 | 1 | 1 | 0 | 4 | 1 | | 0 | 109 | 0 | 1 | [Hemoptysis](https://en.wikipedia.org/wiki/Hemoptysis) after coughing, bronchitis | | |
| 5 | 74 | m | 6 | 6 | 4 | 0 | 0 | 0 | 1 | | 0 | 16 | 0 | 1 | Hemoptysis, hemangioma trachea | | |
| 6 | 65 | f | 3 | 3 | 3 | 0 | 0 | 3 | 1 | | 0 | 50 | 1 | 1 | Colitis ulcerosa with GI bleeding | | |
| 7 | 78 | m | 4 | 4 | 3 | 0 | 0 | 2 | 1 | | 0 | 55 | 0 | 1 | Epistaxis, high blood pressure | | |
| 8 | 73 | m | 4 | 5 | 3 | 3 | 2 | 2 | 2 | | 0 | 92 | 1 | 1 | Epistaxis | | |
| 9 | 74 | f | 5 | 4 | 1 | 0 | 0 | 3 | 1 | | 0 | 19 | 0 | 1 | Recurrent epistaxis | | |
| 10 | 79 | m | 5 | 5 | 3 | 3 | 0 | 2 | 1 | | 1 | 255 | 2 | 1 | Secondary bleeding after tooth extraction | | |
| 11 | 81 | m | 5 | 5 | 3 | 0 | 0 | 1 | 2 | | 1 | 80 | 2 | 1 | Epistaxis after fall, inpatient treatment | | |
| 12 | 85 | m | 4 | 4 | 2 | 2 | 0 | 3 | 1 | | 1 | 413 | 1 | 1 | Spinal bleeding after peridural catheter, obstructive ileus | | |
| 13 | 75 | m | 4 | 5 | 5 | 5 | 4 | 3 | 3 | | 2 | 22 | 5 | 1 | Recurrent GI bleedings, jejunal telangiectasia | | |
| 14 | 81 | m | 4 | 5 | 1 | 1 | 0 | 3 | 1 | | 1 | 1011 | 3 | 1 | Epistaxis after manipulation | | |
| 15 | 69 | m | 1 | 5 | 1 | 0 | 0 | 3 | 1 | | 0 | 66 | 0 | 1 | Hematuria, urinary infection | | |
| 16 | 82 | m | 5 | 5 | 3 | 0 | 0 | 2 | 1 | | 1 | 26 | 3 | 1 | Malaena | | |
| 17 | 87 | m | 6 | 5 | 1 | 0 | 0 | 1 | 1 | | 1 | 66 | 7 | 1 | Recurrent GI bleedings, sigma diverticulosis | | |
| 18 | 81 | f | 5 | 5 | 3 | 0 | 0 | 2 | 1 | | 0 | 478 | 0 | 1 | Hematuria, renal carcinoma | | |
| 19 | 78 | m | 2 | 3 | 3 | 1 | 4 | 3 | 1 | | 1 | 19 | 2 | 1 | GI bleeding, telangiectasia, colon carcinoma | | |
| 20 | 82 | m | 7 | 7 | 1 | 0 | 0 | 4 | 1 | | 0 | 22 | 0 | 1 | Intraabdominal bleeding | | |
| 21 | 73 | f | 3 | 4 | 3 | 0 | 1 | 3 | 1 | | 0 | 10 | 0 | 1 | Delayed bleeding at access site | | |
| 22 | 72 | f | 5 | 4 | 3 | 3 | 3 | 0 | 1 | | 1 | 49 | 4 | 0 | Epistaxis, Morbus Osler | | |
| 23 | 90 | f | 6 | 5 | 3 | 3 | 2 | 0 | 1 | | 1 | 2 | 2 | 1 | Peranal hemorrhage | | |
| 24 | 78 | f | 9 | 5 | 5 | 3 | 3 | 3 | 1 | | 0 | 91 | 1 | 1 | Angiodysplasia in stomach, small and large intestine | | |
| 25 | 86 | m | 6 | 5 | 3 | 3 | 3 | 3 | 1 | | 0 | 131 | 1 | 1 | Bloody diarrhea, colonic polyps | | |
| 26 | 82 | m | 3 | 4 | 1 | 1 | 0 | 3 | 1 | | 0 | 233 | 0 | 1 | Hematuria, prostate tumor | | |

Twenty-six patients had bleeding events after left atrial appendage closure (LAAC). The anticoagulant therapy (ACT) is coded as follows: 0 = none, 1 = mono antiplatelet therapy, 2 = dual antiplatelet therapy, 3 = (novel) oral anticoagulation, 4 = (novel) oral anticoagulation plus antiplatelet therapy. ACT pre LAAC is at first visit, post LAAC at 1 year following LAAC. Abbreviateions: GI = gastrointestinal; RBCT = 1 unit red blood cell transfusion.

**Supplemental Figure 2.** Bar graph showing the anticoagulant therapy and the CHA_2_DS_2_-VASc score at the time of stroke before left atrial appendage closure (LAAC). 9 of the 14 patients (64%) with stroke before LAAC were on antiplatelet (APT), dual antiplatelet (DAPT) or without anticoagulant therapy; 4 were on vitamin K antagonist (VKA) and 1 had a non vitamin K dependent oral anticoagulation (NOAC).
